# Enhancing Crop Yield Prediction Utilizing Machine Learning on Satellite-Based Vegetation Health Indices

**DOI:** 10.3390/s22030719

**Published:** 2022-01-18

**Authors:** Hoa Thi Pham, Joseph Awange, Michael Kuhn, Binh Van Nguyen, Luyen K. Bui

**Affiliations:** 1 School of Earth and Planetary Science, Spatial Science Discipline, Curtin University, Perth 6102, Australia; thihoa.pham@curtin.edu.au (H.T.P.); M.Kuhn@curtin.edu.au (M.K.); 2 Faculty of Surveying, Mapping and Geographic Information, Hanoi University of Natural Resources and Environment, Hanoi 100000, Vietnam; 3 Geodetic Institute, Karlsruhe Institute of Technology, Engler-Strasse 7, D-76131 Karlsruhe, Germany; 4 Geology Faculty, Hanoi University of Natural Resources and Environment, Hanoi 100000, Vietnam; nvbinh@hunre.edu.vn; 5 Faculty of Geomatics and Land Administration, Hanoi University of Mining and Geology, Hanoi 100000, Vietnam; buikhacluyen@humg.edu.vn

**Keywords:** crop yield prediction, vegetation condition index (VCI), thermal condition index (TCI), independent component analysis (ICA), principle component analysis (PCA), machine learning

## Abstract

Accurate crop yield forecasting is essential in the food industry’s decision-making process, where vegetation condition index (VCI) and thermal condition index (TCI) coupled with machine learning (ML) algorithms play crucial roles. The drawback, however, is that a one-fits-all prediction model is often employed over an entire region without considering subregional VCI and TCI’s spatial variability resulting from environmental and climatic factors. Furthermore, when using nonlinear ML, redundant VCI/TCI data present additional challenges that adversely affect the models’ output. This study proposes a framework that (i) employs higher-order spatial independent component analysis (sICA), and (ii), exploits a combination of the principal component analysis (PCA) and ML (i.e., PCA-ML combination) to deal with the two challenges in order to enhance crop yield prediction accuracy. The proposed framework consolidates common VCI/TCI spatial variability into their respective subregions, using Vietnam as an example. Compared to the one-fits-all approach, subregional rice yield forecasting models over Vietnam improved by an average level of 20% up to 60%. PCA-ML combination outperformed ML-only by an average of 18.5% up to 45%. The framework generates rice yield predictions 1 to 2 months ahead of the harvest with an average of 5% error, displaying its reliability.

## 1. Introduction

Accurate crop yield predictions improve decisions about planning effective crop management, allocating government resources, and preparing aid distributions, imports, and exports of agricultural products, see, e.g., [1,2,3,4]. However, yield estimates are challenging due to complex interactions between crop growth and yield-influencing natural factors, such as weather [5,6,7], soil conditions [7,8], disease [9], and anthropogenic factors such as irrigation, fertilizers, tillage, rotation, and seed varieties [9]. Although some crop yield models estimate the yield reasonably well for subregions, e.g., wheat [10,11,12,13,14,15,16,17,18], rice [19,20,21], potato [22,23], soybean [3,4,24,25], maize [26,27,28,29], corn [25,30,31,32], cotton [33], barley [15,17,34], cereal [35], coffee [36], canola [15,37], and sugarcane [17], better performance for yield prediction is still desirable [17].

The two most used approaches for yield prediction are biophysical modelling (see, e.g., [38,39,40,41,42,43]) and empirical regression-based modelling [44]. The former is based on physical relationships between plants and the environment, and they adopt mathematical formulation to derive the accumulated biomass from meteorological data, such as daily temperatures, radiation levels, and rainfall amounts. Some biophysical models, e.g., World Food Studies (WOFOST; [38]), Erosion Productivity Impact Calculator (EPIC; [39]), the Crop Environment Resource Synthesis (CERES; [40]), the Agricultural Production Systems Simulator (APSIM; [41]), and The Decision Support System for Agrotechnology Transfer (DSSAT; [42,43]), require more detailed information about the environment to be incorporated into the modelling procedure. These biophysical crop models generally demand numerous inputs to run and use many assumptions, e.g., [10,17,43]. In contrast, the empirical regression-based models are based on data-driven statistical techniques requiring fewer input data types and fewer assumptions. They use real, on-field, and within-season data to determine empirical relationships between crop yields and yield-influencing variables [17]. Empirical regression-based models have thus recently received more attention from researchers than biophysical models, e.g., [17,44,45,46].

Conventional statistics can determine the regression model for calculating yearly crop productivity if the historical annual crop yield and the vegetation condition index (VCI), as well as the thermal condition index (TCI) time series during the same period, are available, see, e.g., [11,22,32,47,48,49]. This approach requires a good understanding of the relationship between the annual crop yield (dependent variable) and the VCI and TCI data (independent variables), and this is often not satisfied in practice. Assumptions about the relationship are consequently used for creating the models [11,21,22,32,47,48,49]. On the contrary, ML is based on a learning approach that can develop models without any assumption regarding the distribution and interconnections of input variables [50]. Machine learning models outperform traditional regression models in terms of the self-learning process [51] to determine the relationship between the historical annual crop yield (responses) and VCI/TCI data (predictors). However, they require more input data than the conventional regression methods because it is impossible to make predictions well into the future if data patterns have never been seen before.

The performance of ML strongly depends on the availability of historical crop yield data (responses) and yield-impacted variables (predictors). The historical crop production data can be obtained from the Food and Agriculture Organization of the United Nations (FAO, http://www.fao.org/home/en/) or national and regional reports. The yield-impacted factors are generally related to the environment and often rely on satellite-based data that are timely, repeatable, and continuous [52]. Satellite data are cheaper, have higher coverage, and are more accessible than in situ data [21]. Therefore, the rapid development of satellite technology coupled with modern ML methods has recently driven the empirical approach to become more efficient and hence is currently the preferred approach (see, e.g., [3,4,10,11,12,14,15,16,17,18,19,20,22,23,24,27,28,31,32,33,34,36,44]).

Although various satellite-based products (e.g., leaf area index (LAI), green area index (GAI), vegetation index (normalized difference vegetation index—NDVI, enhanced vegetation index—EVI), temperature-vegetation dryness index (TVDI), soil adjusted vegetation index (SAVI), vegetation health index (VCI and TCI), and meteorological parameters) have been used to forecast crop yield in the studies mentioned above, there exists no empirical evidence about which data best predict the crop yield. However, VCI and TCI have two theoretical advantages. Firstly, they are weather-related components of the environment in each ecosystem, and therefore indicate the cumulative weather effects on the annual crop yield fluctuation around the trend [11]. Therefore, if the predictors are VCI/TCI, the responses will be yield deviations from the trend. This approach thus avoids much input information such as environmental factors determining levels of crop yield stability (e.g., climate, ecosystems, soils, and topography; [53]) and technology-related variables shaping a long-term steady yield change (e.g., fertilizers, pest and disease control, hybridization, mechanization; [11]). Secondly, they have a high temporal resolution (weekly) that provides insight into near real-time crop growth. Moreover, their performance as predictors has been confirmed in different crop yield forecasts at various locations, see, e.g., [11,22,49,53].

Even though crop yield models have been developed based on ML methods coupled with VCI/TCI data as discussed above, two challenges remain:

(1) General methods that divide large agricultural regions into subregions that employ separate yield prediction models instead of a one-fits-all approach are lacking. Existing models are generally developed based on resolutions of VCI/TCI data or regional spatial coverage. On the one hand, although VCI/TCI resolution based models offer more detailed crop production forecasts, they have very low feasibility in practice owing to the lack of historical time series of crop yields on VCI/TCI data scales. On the other hand, in theory regional-scale training models are less accurate but are still commonly used in practice (e.g., [3,4,10,11,12,13,14,15,16,17,18,19,20,22,23,24,25,27,28,29,30,31,32,33,34,35,36,37,44]). Using only a one-fits-all prediction model for an agricultural region with spatially varying parameters that influence yield may not well represent some subregions within this area; the model will provide information based only on the “average” conditions. One way of dealing with this issue is to split the region into smaller subregions and use different prediction models for each subregion. In other words, the region should be divided into subregions for which crop yield data are available, and the yield-influencing factors have the same spatial behavior. This issue has not been addressed when forecasting crop yield based on ML methods integrating VCI/TCI products to the best of our knowledge. This shortcoming has been mentioned in other approaches associated with building crop yield models. For example, [24] argued that one of the main challenges of using satellite and weather data as proxies to forecast yield at regional levels is that the crop field boundary and crop-specific layers are not available. Similarly, [17] pointed out that the concept of an ideal spatial domain coverage for modelling approaches should be evaluated.

(2) Techniques to handle redundant VCI/TCI data when using nonlinear ML are lacking: In practice, VCI/TCI data for consecutive weeks have linear correlations in practice [22], and as such, there are redundant data when models are being trained that adversely affect the resulting models [54]. Few studies have addressed this issue by condensing weekly TCI and VCI dataset into smaller numbers of principal components used as predictor variables (e.g., [22,55,56,57]). However, they only used principal component analysis (PCA; [58]) integrated with the linear ML method, i.e., the so-called principal component regression method (PCR; [21,22,55,56,57]). Yield-influencing data and crop production information may have a *nonlinear* relationship in some cases (e.g., [15,59]). There is, therefore, a need for incorporating PCA with nonlinear ML methods, that is, for extending the PCR approach.

This study proposes a framework that tackles the two issues above: (i) the use of a higher-order statistical method of spatial independent component analysis (sICA) to split regions into subregions with uniform VCI/TCI patterns before training the models, (ii) determining the best crop yield prediction models by comparing the performances of the PCA-ML method and the ML-only method, and (iii) employing the best model from (ii) above to analyse the effectiveness of splitting a region into subregions. The framework’s strengths and limitations are assessed based on predictive models of rice yield for subregions in Vietnam from 1995 to 2019.

## 2. The Proposed Crop Yield Prediction Framework

**Figure 1:**
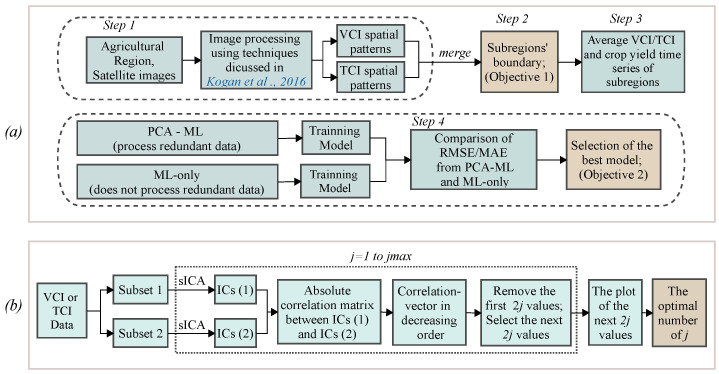
Flowcharts for (**a**) the proposed crop yield prediction framework and (**b**) determining the optimal numbers of the spatial independent components of the VCI/TCI data.

The proposed four-step framework for enhancing crop yield predictions based on VCI/TCI indices, sICA, and PCA coupled with ML methods is summarized in Figure 1a and described in this section.

### 2.1. Step 1: Generation of VCI and TCI Data from Satellite Data

The agricultural region boundaries are first extracted from administrative maps and then integrated with satellite images to determine the VCI/TCI data for the region.

The VCI and TCI are generated from the normalized difference vegetation index (NDVI) and brightness temperature (BT), respectively, [11]. The NDVI presents the amount of green vegetation in an area [11,60]. The BT is calculated from (thermal) infrared channels, thus showing the thermal vegetation conditions [11,60].

The NDVI and BT have been represented with two components: (i) the spatial difference between the productivity of ecosystems, which is considered as the level of NDVI/BT values, and (ii) the weather-related variations in each ecosystem, which is the ratio of the difference between the actual and the minimum values and the range of NDVI/BT. The first one, called the ecosystem components, relates to long-term environmental variables such as climate, soils, topography, and landscape. The second one presents a short-term weather component [11,22,47]. The weather components in NDVI and BT are the VCI and TCI, respectively, [47,60]. The VCI, a proxy for the chlorophyll and moisture contents of the vegetation canopy, characterizes plant greenness and vigor. In contrast, the TCI describes thermal conditions [22,32] and moisture availability through near-surface radiation and aerodynamic shapes [61].

The VCI and TCI are considered annual weather-related fluctuations of the NDVI and BT from their climatologies. As a yearly crop yield deviation from a long-term yield trend is often dominated by weather changes [11], it is strongly related to VCI/TCI data. This relationship is the main reason for adopting the VCI and TCI as predictors in developing crop yield forecasts.

The VCI and TCI are generated from satellite images via three multilayered steps: (1) the NDVI is calculated from the ultraviolet-visible (VIS), and near-infrared (NIR) reflectance, and the BT is generated from infrared emissions; (2) the high-frequency noise is removed from the NDVI and BT; and (3) the VCI and TCI are estimated from the NDVI and BT, respectively, as follows [60]:

(1) $$\mathrm{VCI}=\frac{NDVI-NDVImin}{NDVImax-NDVImin}\times 100\%,$$

(2) $$\mathrm{TCI}=\frac{BTmax-BT}{BTmax-BTmin}\times 100\%,$$

where NDVImin and NDVImax are the minimum and maximum values of the NDVI, respectively; BTmin and BTmax are the minimum and maximum values of the BT, respectively. The TCI and VCI indices are scaled to range from 0 (severe vegetation stress) to 100 (favourable conditions for vegetation growth) [60]. Details of the procedure are presented in [60].

It is worth noting that the two indices are derived by eliminating the long-term components related to climate from the NDVI and BT [48,60]. Their time series data should thus span over more than 30 years to meet the demand of studying climatic patterns [62]. Their temporal resolution is also more substantial than their spatial resolution because weather characteristics in NDVI/BT have a low spatial variation and the crop state rapidly changes during the growing season. Therefore, the requirements of high temporal resolution and more extended 30-year time series lead the VCI/TCI data from the National Atmospheric and Oceanic Administration (NOOA) to be selected. The near real-time weekly VCI/TCI data from 1981 to date already existed in a gridded form. This study downloaded VCI/TCI sub-datasets via the link: https://www.star.nesdis.noaa.gov/smcd/emb/vci/VH/vh_ftp.php (accessed on 9 January 2021).

### 2.2. Step 2: sICA-Based Determination of Subregions

The independent component analysis (ICA) [63] is employed to statistically determine the spatial independent components of VCI/TCI as follows:

(3) X(t,s)=Aj(t)Sj(s),

where X(t,s) is a gridded time series of VCI/TCI, *t* is the time, *s* represents the grid points, and *j* is the number of independent components (ICs) equivalent to the quantity of different spatial patterns of VCI/TCI. The time series of X(t,s) is decomposed into spatial Sj and temporal Aj models. The rows of *S* represent spatial patterns being statistically as independent as possible, and the columns of *A* are their corresponding temporal evolutions. In this case, the ICA technique is the so-called spatial ICA (sICA) [64].

The resulting sICA-based patterns are subregions that have the same spatial characteristic. The outcome models are statistically independent and can be separately analyzed without considering other models [64].

Unfortunately, the number of independent models (*j* value) is unknown because ICA is a blind source separation method. Therefore, the most crucial consideration is to find out the reasonable number of *j*. This number is then used to determine spatial patterns of VCI/TCI based on Equation (3). VCI spatial patterns differ from those of TCI. Therefore, subregions are generated by merging the two spatial patterns in the final step.

The optimal number of *j* is determined based on the ICA-by-blocks method [63] as recapped in Figure 1b and described below step-by-step.

Firstly, the VCI/TCI time series is divided into two subsets of approximately equal size, ensuring they represent the whole data matrix. Secondly, Equation (3) is applied with the value of *j* (the number of ICs) changing from 1 to jmax for each subset. Thirdly, with a particular value of *j* (1<j<jmax), the square matrix of order 2j for absolute correlations between ICs generated from two subsets is determined. As a result, the jmax matrices of correlation are generated. Fourthly, the correlation matrices are vectorized in decreasing order. Fifthly, the first 2j values (the correlation of an IC with itself) of the correlation vector are removed, and then the next 2j values are selected. Finally, the selected value vector is plotted (only every first or second point of the chosen vector is plotted because of the duplicate values of IC between two subsets). The resulting figure is then used to determine the optimal number of spatial components. If *j* is the proper value, the correlation between all equivalent ICs in two subsets will be close to 1. In contrast, the redundant ICs will contain an unusual noise, and they will be significantly less correlated with all ICs from the other subset. Therefore, the optimal number of spatial components is defined as the point at which all the correlations are relatively high.

The value of jmax should exceed the expected optimal number of *j* (number of ICs) and is therefore selected based on the experiment. For example, the value of jmax is increased until the optimal number of *j* is stable.

### 2.3. Step 3: Preparation of Predictor and Response Variables

#### 2.3.1. Determining the Average VCI/TCI Time Series (Predictor Data) for Each Subregion

Missing VCI/TCI gridded time-series values are first replaced by long-term mean values in a year’s corresponding time. Then, the average VCI/TCI time series for each subregion (predictor data) is generated in the form of a spatially averaged VCI/TCI time series. Finally, the year is defined based on the crop season, beginning from the first week after the plant is harvested and ending at the last week of the harvest season.

#### 2.3.2. Determining Detrending Average Crop Production Time Series (Response Data) for Each Subregion

In general, the annual crop yield depends on environmental conditions, applied technology, and weather factors. The environment, that is, the climate, ecosystems, soils, and topography, is a stable factor and determines the level of crop productivity [53]. Technology (e.g., fertilizers, pest/disease control, hybridization, and mechanization) influences the long-term steady yield change. As a result, environmental and technological factors determine the trend of crop yield. Finally, the weather factor dominates a short-term weather-related annual crop yield fluctuation around the trend during the year’s growing season. Crop productivity often exceeds the trend if the weather is more favourable for crop growth. In contrast, yields often dip below the trend if less favourable weather conditions occur [11].

As mentioned in Section 1, the VCI/TCI represents the cumulative weather effects on annual crop yield fluctuation around the trend. Therefore, if the predictor is the VCI/TCI, the response dataset will be the yearly detrending average crop yield time series. Thus, in this step, the annual crop productivity time series is first calculated as the spatial average values (Yi, where the index *i* refers to the *i*th year) for a separate subregion derived in step 2. The crop yield Yi is then separated into two components as [10,32]:

(4) Yi=Ti+dYi,

where Ti is a level and long-term yield trending component corresponding to ecosystem components and agricultural technology improvements and dYi is a short-term weather-related yield variation around the trend.

#### 2.3.3. Splitting Predictor/Response Data into Training and Test Datasets

Predictor and response datasets are split into training and test sets based on a stratified sampling strategy ([65] p. 51), ensuring they represent the data at hand. Firstly, the response dataset is sorted into smaller homogeneous subgroups called strata with shared attributes or characteristics. Secondly, the response training and test datasets are generated by gathering the proportional data (e.g., 80% for the training set and 20% for the test set) in all subgroups. Finally, the predictor training and test datasets are produced for the same period as the response dataset. In this way, the model will be trained and tested based on a generalized sample. As a result, validation and test accuracies will better represent model performances.

### 2.4. Step 4: Development of Crop Yield Prediction Models

Separate crop yield models are built for each subregion and growing season. For each case, an ML method is adopted for developing two crop yield models following two options, one that incorporates PCA (PCA-ML) and one that does not incorporate PCA (ML-only). Finally, the model that performs better is selected as the final model.

In the PCA-ML method, the role of PCA is to rotate the predictor data in such a way as to align the directions in which the data spreads out the most with the principal axes, reducing the data dimensionality while keeping the variance as close to the original data as possible [66]. Hence, the PCA approach benefits by eliminating the linear correlations in the predictor data leading to the PCA-ML combination, which generate better results than when ML-only is use.

Two common statistical indicators, the mean absolute error (MAE) and root mean square error (RMSE), are used for model assessment. The RMSE highlights significant errors because they are squared before they are averaged, whereas the MAE clarifies the average error. The RMSE/MAE is expressed as a percentage by dividing each indicator by the yield’s mean to indicate how good the predicted yield is relative to the actual yield.

#### 2.4.1. Training the Model

Many ML algorithms have been adopted for developing crop yield forecasting models in different studies. There are no specific conclusions regarding the best model, but some ML models are employed more than others in practice. The commonly used models are the random forest, artificial neural networks (ANN), linear regression, and gradient boosting tree models [44]. The ANN is the most commonly used algorithm [44]. However, in our view, the ANN method is unsuitable in a framework that aims to develop a model at a subregional scale because it does not work well with limited data ([65] p. 26). Therefore, the framework proposes some commonly used regression ML methods for training predictive yield models, such as linear regression, support vector machine, and decision tree methods.

Each ML algorithm comprises different methods. For example, linear regression includes the linear and boost linear methods. The support vector machine is developed with kennel linear, quadratic, cubic, and Gaussian functions. The decision tree has the decision tree, decision ensemble boost tree, and ensemble bagged tree methods. The detailed theoretical background of these ML methods are presented in, e.g., ([65] pp. 145–179), [67].

The performance of ML algorithms depends on their hyperparameter settings ([65] p. 28). Each ML method has a corresponding hyperparameter type that performs differently for different datasets of predictors and responses. In essence, hyperparameter tuning uses a cross-validation approach for exploring an excellent pattern in parameter spaces. A validation dataset is held back from the training dataset to estimate the model’s performance while the model’s hyperparameters are tuned ([65] p. 30). It should be noted that the VCI/TCI predictors have been available from 1982 to the present, or 39 years. The maximum value of responses (detrending crop yield) is thus 39. This number is not significant enough so that the leave-one-out cross-validation is considered the best method for tuning the model hyperparameters in the proposed framework.

#### 2.4.2. Testing the Models

The model testing is separated from the model training. The error on the test set, called the generalization error, shows how well the model performs in instances it has never seen before.

## 3. A Case Study of Vietnam’s Rice Production

### 3.1. Vietnam: Background

As the second-largest rice-producing country globally (https://vietnaminsider.vn/exceeding-thailand-vietnam-becomes-worlds-2nd-largest-rice-exporter/), Vietnam plays a significant role in international food security. However, although rice production has overall increased in the past decades, it has fluctuated significantly. Therefore, in this section, the proposed framework will be evaluated in predicting Vietnam’s rice production.

**Figure 2:**
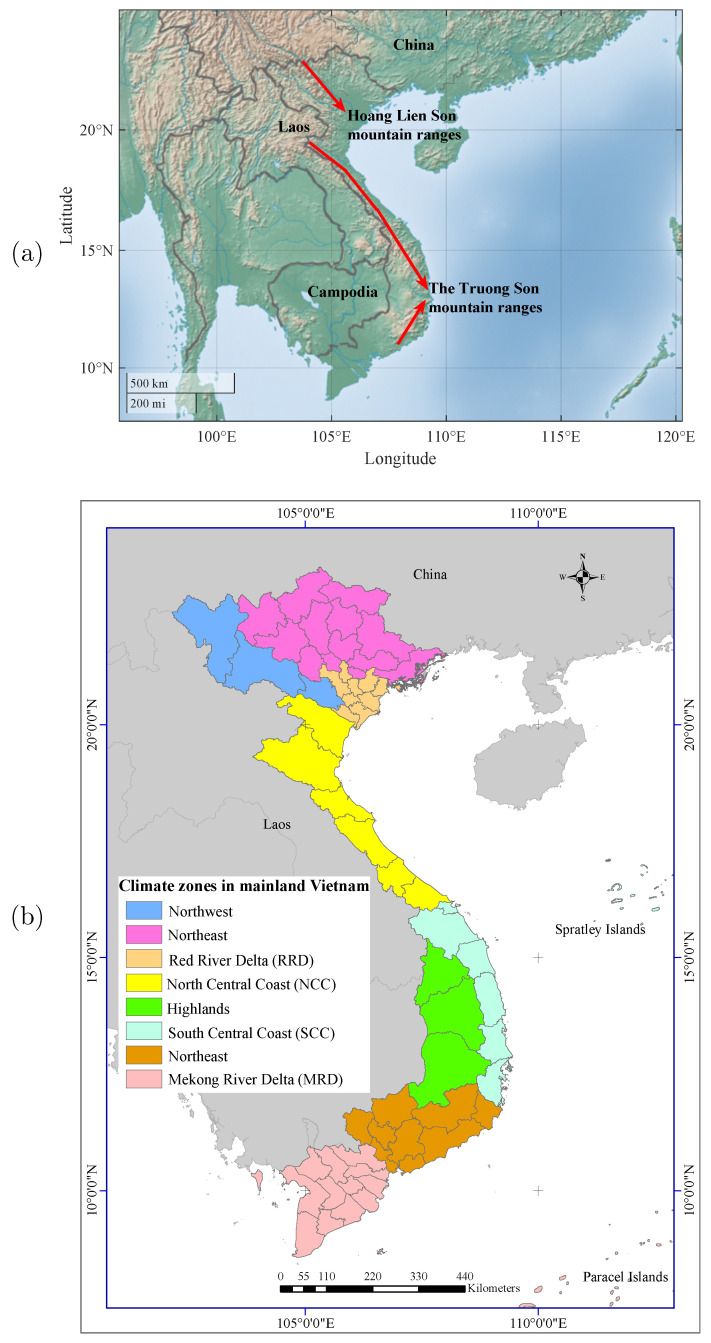
(**a**) The Hoang Lien Son mountain and the Truong Son mountain ranges in Vietnam; (**b**) eight climate zones in mainland Vietnam: Northwest, Northeast, RRD, NCC, SCC, Highlands, Southeast, and MRD.

Vietnam’s climatic characteristics are quite diverse and vary from subregion to subregion across the country, while the northern part experiences subtropical monsoon and has four distinct seasons (spring, summer, autumn, and winter), the central and southern regions experience tropical monsoon and have two seasons (rainy and dry). The climate is strongly dominated by the southwest (summer) monsoon from May to October and the northeast (winter) monsoon from November to April [68]. Besides the geographic location, Vietnam is also affected by varied topographic conditions. For example, the Hoang Lien Son mountain range (Figure 2a) in northwest Vietnam divides the northern mountainous region into western and eastern parts. The Truong Son (Annamite) mountain ranges (Figure 2a) stretch along the western border and end at the South Central Coast, making the coastal zone hotter and the differences between the northern and southern climates more pronounced. The Central Highlands borders the lower part of Laos and northeastern Cambodia. It lies on a series of contiguous plateaus and is surrounded by high mountain ranges, such as the South Annamite Range (Figure 2a); this causes its year-round climate to be colder than that of Vietnam’s coastal regions.

The changing climatic characteristics based on geographic location and topographic conditions have resulted in eight distinct subregional climate zones across the Vietnamese mainland (Figure 2b): Northwest, Northeast, Red River Delta (RRD), North Central Coast (NCC), South Central Coast (SCC), Highlands, Southeast, and Mekong River Delta (MRD).

### 3.2. Generation of VCI and TCI Data from Satellite Data (Step 1)

Before playing the role of predictor, the time series are used to determine Vietnam’s subregions with the same VCI/TCI spatial characteristics. For this purpose, the VCI/TCI time series should be collected as long back in time as possible because the longer the time over which the VCI/TCI data have been gathered, the better the results for the subregions. Therefore, VCI/TCI data with the spatio-temporal resolution of 4 km and a 7-day composite from January 1982 to December 2020 are downloaded for Vietnam’s mainland directly from NOAA (cf. step 1 in Section 2), and a sub-dataset is extracted.

When playing the role of the predictor in the training yield forecasting model, the VCI/TCI data are selected for the same period as the rice production dataset.

### 3.3. sICA-Based Determination of Subregions (Step 2)

#### 3.3.1. The Number of Independent Components of sICA

**Figure 3:**
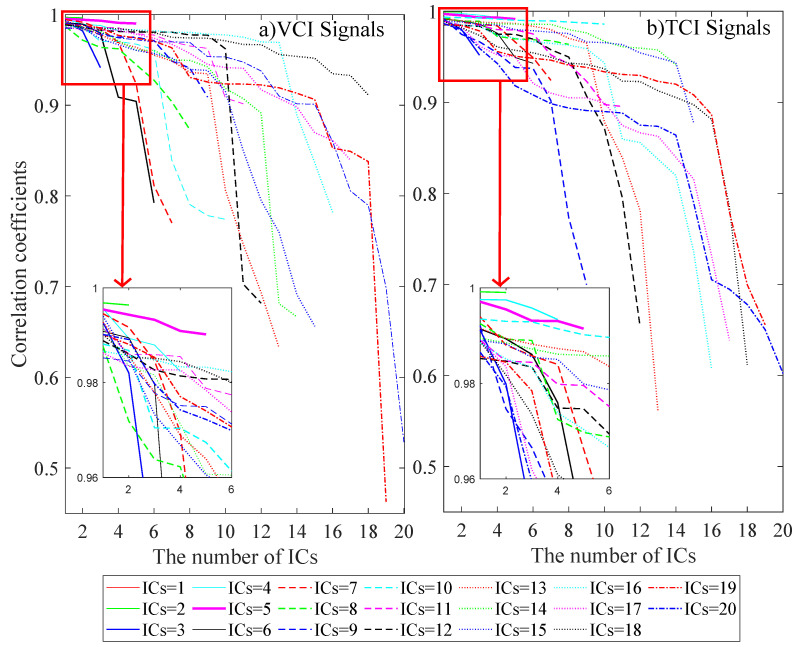
The signal-correlation graph for the VCI and TCI data.

The ICA-by-Blocks model (cf. step 2 in Section 2) is applied separately for the VCI and TCI datasets. The correlation between the ICs of the two subsets (*p*-value < 0.05) is presented in Figure 3a,b for VCI and TCI, respectively. It can be seen that the correlations are close to 1 for ICs ranging from 1 to 5 for both VCI and TCI signals. After 5 ICs, the correlation reduces progressively when the number of ICs increases to 6 or 7 and up to 20 ICs. The maximum investigated number of 20 ICs is significant enough because when adding more than 5 ICs, all the correlations between the ICs of the two subsets are much lower. The optimal number of ICs for VCI and TCI is thus set to five in this study.

#### 3.3.2. Spatial Patterns of the VCI and TCI

**Figure 4:**
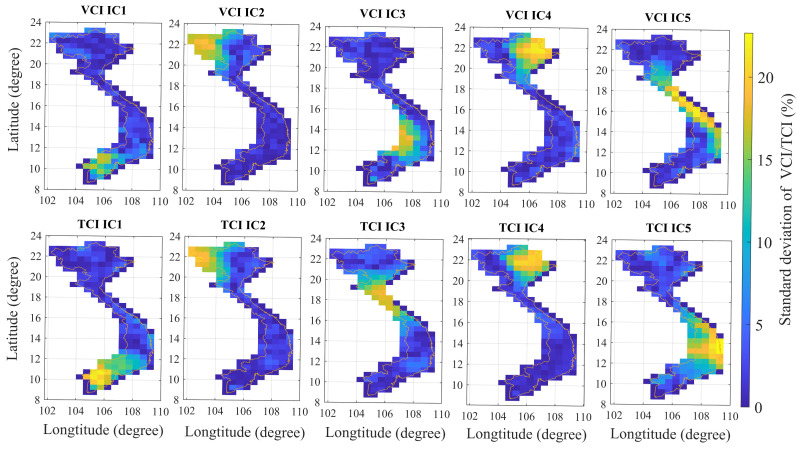
VCI (top) and TCI (bottom) spatial patterns in mainland Vietnam.

Five ICs are utilized to determine the independent spatial patterns of VCI/TCI based on the sICA technique. Each IC represents a separate zone with the same spatial characteristic ranges of VCI/TCI (Figure 4 and Table 1). The results show that IC2 generated from VCI products and IC2 from TCI have similar patterns. Likewise, IC4 from VCI and IC4 from TCI products share similar pattern (i.e., Figure 4 VCI (IC2) and TCI(IC2) as well as VCI(IC4) and TCI (IC4) have similar patterns). All the remaining ICs are distinct.

#### 3.3.3. Resulting Subregions Based on Combining the Spatial Patterns of VCI and TCI

From Figure 4, subregions are generated as shown in Table 2. Although the Northeast and RRD have the same spatial patterns of VCI/TCI, the rice yield prediction mode for each region should be built separately. The RRD is one of two delta areas in which most of the land is devoted to rice cultivation. In contrast, the Northeast area is primarily mountainous and has small plains used for rice cultivation between the north and its flat regions that extend toward the coast and the south.

In the following steps, the process of developing models is therefore conducted for each subregion of rice crops, such as the Northwest, Northeast, RRD, NCC, SCC, Highlands, Southeast, and MRD. In addition, the one-fits-all model for the entire country of Vietnam is also built because it plays a reference role in measuring the effectiveness of splitting the region into subregions.

### 3.4. Preparation of Predictor and Response Data for Each Subregion (Step 3)

#### 3.4.1. Detrending Average Rice Production Time Series (Response Data) for Subregions

In Vietnam, the rice-growing seasons are categorized into the Winter–Spring, Fall–Winter, and Summer–Autumn seasons. The seasonal rice yields (averages over all of the provinces) from 1995 to 2019 are collected from the General Statistics Office of Vietnam via the link: https://www.gso.gov.vn/Default20en.aspx?tabid=491, accessed on 15 January 2021.

The seasonal rice yield of a particular subregion is first computed as a spatial mean value, and the long-term trend is then removed to generate the de-trended average rice production time series (response data).

**Figure 5:**
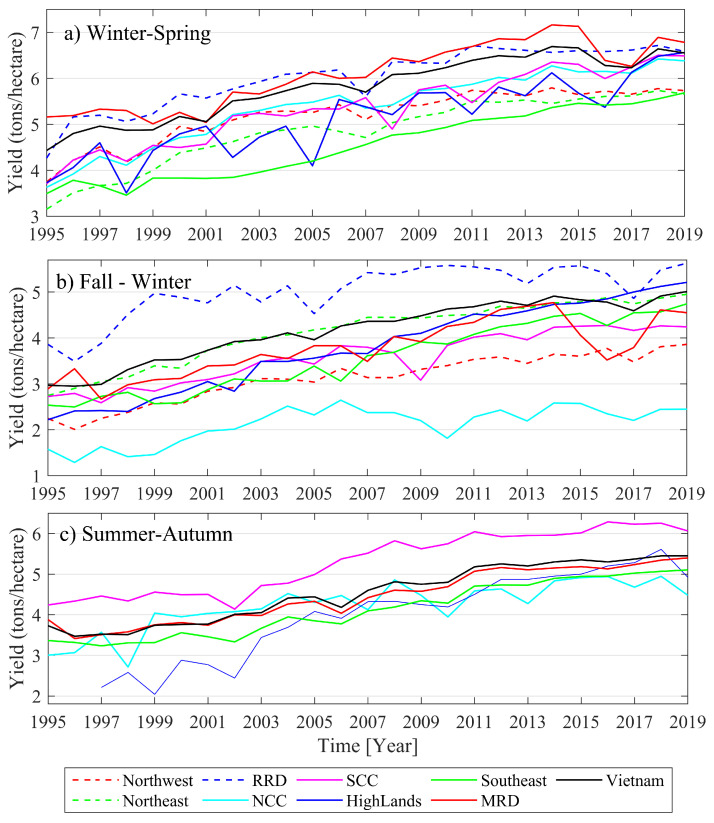
Average rice production time series for subregions listed in Table 2 in mainland Vietnam.

Five subregions in Table 2, namely, the NCC, SCC, Highlands, Southeast, and MRD, have three rice seasons per year (Winter–Spring, Fall–Winter, and Summer–Autumn), and the remaining three subregions, namely, the Northwest, Northeast, and RRD, have only two rice seasons per year (Fall–Winter and Winter–Spring). All of the annual rice yield time series are for the period 1995 to 2019, except the Summer–Autumn rice dataset in the Highlands, which is for the period 1997 to 2019 (Figure 5). Figure 5 shows that although the average annual rice yields had a trend of increasing, they still experience different magnitudes and variabilities. The median and standard deviations of rice yields vary from subregion to subregion and also from season to season (these data are not shown in Figure 5). There is, therefore, a need to develop rice production models for individual seasons in each subregion.

#### 3.4.2. Average VCI/TCI Time Series (Predictor Data) for Subregions

Like the rice production time series, the average VCI/TCI data has to be generated for each subregion. However, these data were already provided by NOAA via the link: https://www.star.nesdis.noaa.gov/smcd/emb/vci/VH/vh_adminMean.php?type=Province_Weekly_MeanPlot, accessed on 15 December 2020.

The VCI/TCI time series is extracted from 1994 to 2019, which matches the data for rice production from 1995 to 2019. The additional VCI/TCI data for 1994 is necessary because the first rice crop harvested in 1995 was planted in 1994. Each year, although the average rice yield of a particular season is a single value, the average VCI/TCI includes 52 weekly values. Missing values for weeks 37 to 52 in 1994, weeks 2 to 29 in 2004, and weeks 1 to 6 in 1995 are replaced by the long-term mean values of the respective weeks from other years.

#### 3.4.3. A Training Dataset and a Test Dataset for Each Rice Season

The average VCI/TCI weekly time series data derived in Section 3.4.2 are rearranged following the planted time for each rice season. It should be pointed out that the weeks are not in standard calendar years but in years of the harvest season that starts after the previous harvest season and ends in the last week of the current harvest season. The last weeks are thus the rice planting, growing, and harvesting times.

Each response (annual detrended rice yield) has its corresponding predictor dataset. The predictor and response datasets for each rice season are separated into two sets based on the stratified sampling strategy: The training set includes predictors and responses for 20 years, and the test set includes data for the remaining 5 years. However, for the Summer–Winter data for the Highlands, the training set and test set are a 19 year-long time series and a 4 year-long time series, respectively.

### 3.5. Development of Rice Yield Prediction Models (Step 4)

The training and testing datasets derived in Section 3.4.3 are adopted to develop a yield prediction model for each subregion and individual rice season. To evaluate the effectiveness of the proposed framework following the objectives established in Section 1, the process of building models is implemented as follows:

#### 3.5.1. Comparing the Performance of PCA-Ml with ML-Only

**Figure 6:**
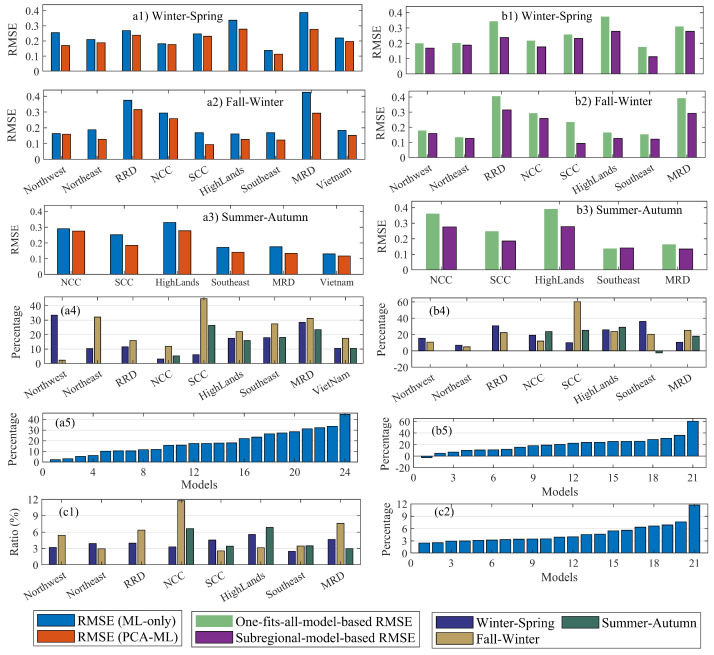
(**a1**–**a3**): The RMSE of the PCA-ML/ML-only-based models in subregions, unit in tons/hectare; (**a4**,**a5**): the improvement of the PCA-ML-based models compared with the ML-only-based models in the subregional groups (**a4**) and it is arranged from the lowest value to highest value (**a5**), unit in percentage; (**b1**–**b3**): the one-fits-all-model-based RMSE and regional-model–based RMSE in subregions, unit in tons/hectare; (**b4**,**b5**): the accuracy improvement of subregional models compared with one-fits-all models in the subregional groups (**b4**) and it is sorted in increasing sequence (**b5**), unit in percentage; (**c1**,**c2**): the ratio of the RMSE and the mean rice production in the subregional groups (**c1**) and is sorted in ascending order (**c2**), unit in percentage.

The ML method mentioned in Section 3.4.1 is employed to build the rice production models. Here, the ensemble boost tree method is selected. Two models are generated for each rice season in each subregion based on this ML method coupled with PCA or not coupled with PCA. The PCA’s contribution is assessed by comparing the test RMSE/MAE of the two models (Figure 6(a1–a5)). It should be noted that different ML methods in Section 3.4.1 can be investigated to choose the optimal one. However, this work is beyond the scope of this paper.

Figure 6(a1–a5) show the upgrade of the PCA-ML-based rice forecasting models compared with the ML-only-based ones. Figure 6(a1–a3) display the tested RMSE of the PCA-ML/ML-only-based models for Winter-Spring, Fall-Winter, and Summer-Autumn, respectively. Figure 6(a4) expresses the improvement by the PCA-ML models are in the subregional groups. In contrast, Figure 6(a5) sorts this information from the lowest value to highest value. Figure 6(a4) shows that for the Northwest, Northeast, and RRD, the left and right columns represent Winter–Spring and Fall–Winter, respectively; the left, middle, and right columns are for Winter–Spring, Fall–Winter, and Summer–Autumn, respectively, for the remaining areas.

The data shown in Figure 6(a1–a3) reveal that the PCA-ML-based models generally outperform the ML-only-based models in all rice seasons and subregions. The PCA-ML’s skill is more transparent with the data in Figure 6(a4,a5), where its effectiveness versus ML-only is measured by dividing the difference between the PCA-ML-based RMSE and the ML-only-based RMSE by the ML-only-based RMSE expressed as a percentage. The PCA-ML-based models’ improvement varies from subregion to subregion and from season to season in the range of 2% to 45%. In general, the average RMSE of the PCA-ML-based models is 0.045 tons/hectare smaller than that of the ML-only-based ones, and the PCA-ML-based models are 18.5% better than the ML-only-based ones (these data are not shown in Figure 6).

#### 3.5.2. Analyzing the Effectiveness of Splitting Regions into Subregions

As the PCA-ML outperforms the ML-only, this step employs the PCA-ML to analyze the effectiveness of splitting the whole of Vietnam into subregions. Firstly, the subregional crop yield models using sICA are trained for rice seasons for each subregion. The test RMSE/MAE generated in this step is called here the subregional-model-based RMSE/MAE. Secondly, the one-fits-all crop yield models are developed for the entire country of Vietnam. Thirdly, one-fits-all outcome models are employed to generate rice yields for the test sets in the subregions. This step calculates the corresponding test RMSE/MAE (called here the one-fits-all-model-based RMSE/MAE). Finally, the improvement of the subregional model versus the corresponding one-fits-all model is assessed by comparing the subregional-model–based RMSE/MAE and one-fits-all-model-based RMSE/MAE. The RMSE/MAE difference (see Figure 6(b1–b5) shows the effectiveness of sICA when splitting an agricultural region into subregions to determine rice yield forecasts in Vietnam.

Figure 6(b1–b3) show the one-fits-all-model-based RMSE (the left column) and the subregional-model–based RMSE (the right column) in each subregion for Winter–Spring, Fall–Winter, and Summer–Autumn, respectively. At the same time, Figure 6(b4) indicates the percentages of improvement of the 21 subregional models compared with the corresponding one-fits-all models in each subregion. These data result from the difference between the subregional-model–based RMSE and the one-fits-all-model-based RMSE divided by the one-fits-all-model-based RMSE. In Figure 6(b4), the left and right columns represent the Winter–Spring and Fall–Winter data, respectively, for the Northwest, Northeast, and RRD. In contrast, the left, middle, and right columns are for the Winter–Spring, Fall–Winter, and Summer–Autumn data, respectively, for the remaining subregions. Figure 6(b5) shows the same information but it is arranged from the smallest to the largest percentage. Generally, the subregional models using sICA outperform the one-fits-all ones (see Figure 6(b1–b3), with the exception of the Summer–Autumn model in the Southeast, which has the same performance as the corresponding one-fits-all model (see Figure 6(b3,b4)). The subregional models’ improvements fluctuate from subregion to subregion and from season to season (see Figure 6(b4)). The improvements are in the range of 5% to 60% (see Figure 6(b5)). The average RMSE of the subregional models is smaller than that of the one-fits-all ones at a level of 0.055 tons/hectare (20%) (these data are not shown in Figure 6).

#### 3.5.3. Analysing the Effectiveness of the Proposed Framework in General

The ratio of the RMSE to the mean rice production is also considered a factor in evaluating the effectiveness of the models in particular and the proposed framework in general. The ratio is shown in subregional groups (see Figure 6(c1)) and in increasing order (see Figure 6(c2)). It changes from subregion to subregion and season to season by approximately 5% (not shown in Figure 6). The lower ratio varies from 2% to 5% in 15 of 21 models (accounting for 71%). The higher values range from 6% to 8% in four models (accounting for 24%) for the Fall–Winter (RRD and MRD) and Summer–Autumn (NNC and Highlands). The highest value of 11.7% is in the Fall–Winter in NNC. In addition, it is worth noting that all models are trained for generating the rice yield for 1 to 2 months before harvest.

Similarly, the improvement of the PCA-ML and subregional models in the MAE matrix shares the same patterns with the RMSE matrix, so the data are not shown here.

## 4. Discussion: Strengths and Limitations of the Proposed Framework

This paper develops a framework for enhancing the accuracy of estimating crop yields using VCI/TCI data and the techniques of sICA and PCA coupled with the ML algorithm. The framework has demonstrated that it can overcome the one-fits-all and redundancy limitations, i.e., sICA technique divides the agricultural region into subregions where yield-influencing factors have the same spatial patterns. At the same time, the combination of PCA and ML algorithms processes flexibly redundant input data.

The proposed framework is applied to generate rice yield prediction models in Vietnam. The outcome reveals the performance of the sICA and PCA-ML methods for enhancing crop yield prediction, which has shown the strengths and limitations of the framework. The significant achieved results of yield predictive models are generally related to the following multiple crucial factors:

*(1) The sICA technique generates optimal subregions having the same spatial pattern of yield-influencing factors, leading to higher accuracy and efficiency in predicting crop yields on subregional scales*: The optimum numbers of subregions are first determined and then used for deriving boundaries to delineate each region. Both processes are completed based on the advanced statistic method, resulting in an excellent theoretical foundation of subregional determination.

Splitting the agricultural region into optimal subregions contributes to the computational simplicity because the models are developed on a larger subregional scale than the VCI/TCI spatial resolution. It also benefits the efficiency of estimating crop yields because the subregions are not randomly determined; rather, they are empirically determined. It ensures that yield-influencing elements have similar behaviours. The spatial average values can be presented for an entire subregion, which is essential for developing a unique model for each subregion.

The theoretical advantages of the new approach to subregional determination have also been confirmed in practice. The case study results show that the VCI/TCI uniform spatial subregions generated based on the sICA method are consistent with the subclimate regions created before by many climate and geographic data.

As boundaries are the foundation for developing subregional models, the efficiency of subregional determination is also validated by the promising subregional rice yield forecasting models achieved. The improvement of the subregional models over the one-fits-all ones is considerable, with the accuracy increasing on average by 20% and up to 60% when using PCA-ML.

This approach for separating subregions can also be applied to other types of input data.

*(2) The combination of the PCA and ML methods deals with redundant data in the VCI/TCI time series, which facilitates estimating yields to become efficient*: Theoretically, the VCI/TCI data may be redundant because they have linear correlations. Practically, ref. [22] proved the existing linear relationship of the VCT/TCI data. The case study also confirms this phenomenon of VCI/TCI data for all subregions in Vietnam (not shown in the paper). The resulting models also reveal the efficiency of PCA. The performance of PCA-ML-based models is generally better than ML-only-based ones for all rice seasons. The accuracy of PCA-ML-based models is better than that of ML-only-based ones at the average level of 18.5% and a maximum value of 45%.

*(3) Integrating sICA and PCA-ML/ML-only for estimating crop yields could generate an excellent crop yield forecasting models’ performance*: The sICA technique first supports optimally dividing the agricultural region into small subregions. The models are then developed for each zone based on a combination of PCA with ML methods, eliminating the influence of collinearity in the yield-influenced data. As the above analyses reveal the role of each model, the integration of sICA and PCA-ML/ML-only makes the proposed framework very methodologically rigorous and feasible in practice, so that the framework successfully overcomes the two limitations in previous works. The framework produces rice yield forecasts for 21 rice yield models in Vietnam at 1 to 2 months before harvest while having minimum requirements for input variables. The ratio between the RMSE and the rice yield’s mean is an average level of 5% (2% to 5% in 15 out of 21 models and 6% to 11.7% for the rest). The achievement can be significant for the Vietnamese government as it would allow early decision making about its rice distribution plan. The result agrees with some other studies. For example, ref. [59] showed that MODIS-NDVI could be employed to forecast crop yields over the Canadian Prairies 1 to 2 months ahead of harvest. Ref. [10] concluded that an empirical NDVI-based regression model could produce winter wheat yield forecasts at the regional (oblast) level in Ukraine 2 to 3 months before harvest. Ref. [57] showed that the wheat yield could be estimated from the VCI index approximately four weeks before harvest time in the United States. Ref. [56] revealed that corn yield could be estimated from the VCI and TCI 2 to 3 months before harvest time with an average 6% error in Haskell County, Kansas, United States. Ref. [11] also developed a model that could derive predicted yields in Australia 1 month ahead of harvest.

Similar results may likely be achieved when applying the framework over other regions and different crops. Apart from the strengths mentioned above, some limitations of our framework still exist:

The PCA contributes to dealing with the linear relationship of the VCI and TCI time series in the theoretical aspect. However, the PCA contribution is not always noticeable in practice. The case study shows that four PCA-ML–based models are not much better (the improvement is less than 6%) than the corresponding ML-only–based model’s performance. There may be more prominent noise in the VCI/TCI data for the four cases, which would affect the maximization of the variance, causing the PCA not to work well.

The VCI and TCI are generated based on satellite images. Thus, their accuracy is dominated by many environmental factors. For example, ref. [69] argued that the VCI does not work well in areas with wet conditions. The case study confirms this assessment. Lower improvement of the PCA-ML models is in regions that have always experienced floods (e.g., NCC; see Figure 6(a4)) and areas having more cloudy days (e.g., Northwest, Northeast, RRD, NNC; see Figure 6(a4)). The PCA method should thus only be an optional approach for better results in some cases. This consideration agrees with some previous studies, such as [21,55,56,57].

In summary, the proposed framework could generate crop yield prediction models on a subregional scale in the contexts of both theory and practice. However, the PCA should only be considered an additional option. The framework’s approach could also develop other predictive regression models that use spatio-temporal data as predictors.

## 5. Conclusions

This study proposes a new framework using sICA, PCA, and ML for enhancing crop yield prediction based on VCI/TCI data. sICA divides an agricultural region into subregions with uniformly spatio VCI/TCI patterns before training the models, and the combination of PCA and ML aims to improve the accuracy of the outcome models compared with the ML-only method. The framework’s performance in the case study, in which high accuracy is achieved in predicting rice yields at the subregional level in Vietnam, suggests that confidence in the proposed framework is warranted. Furthermore, although there is a limitation regarding the weak contribution of PCA in some cases, the proposed framework can nonetheless provide improved predictive yield forecasts. Specifically, the results indicate that:

(1) The proposed sICA technique split the agricultural region into optimal subregions. As a result, the VCI/TCI signals have the same behaviour in each subregion, contributing to computational simplicity and efficiency. The case study in Vietnam proves that sICA can determine the VCI/TCI uniform spatial subregions consistent with subclimate regions created before by many climate and geographic data. The effectiveness of subregion establishment based on sICA is also confirmed by the promising achieved subregional rice yield forecast models, which are better at an average level of 20% and a maximum level of 60% than corresponding one-fits-all models.

In addition, this approach can be applied not only for data from the VCI/TCI but also for other input data.

(2) The PCA can deal with redundant data in the VCI/TCI time series when developing crop yield prediction models in theory. The case study also shows that the skill of PCA-ML-based models is generally better than ML-only-based ones for all rice seasons. The improvement is at an average level of 18.5% and a maximum value of 45%.

Although the PCA technique can contribute ML methods for better results if a linear relationship exists in VCI/TCI time series in theoretical considerations, the PCA significance is only shown clearly in 17 of 21 rice yield prediction models in the case study. The benefits of PCA in the four remaining PCA-ML-based models were not significant, with an improvement in accuracy of only 6%. This means that the PCA should be an optional approach and that incorporating the PCA with ML methods facilitates estimating yields and is more flexible.

(3) The integration of sICA and PCA-ML for crop yield estimation based on VCI/TCI data has excellent advantages. For example, the ratio between the RMSE and the mean rice yield has an average level of 5%. In addition, the combination of sICA and PCA-ML produces rice yield forecasts in Vietnam 1 to 2 months before harvest while having minimum requirements for input variables. This facilitates early decisions regarding the distribution of agricultural products and food security.

The validation of the selected case study of Vietnam affirms the reliable performance of the proposed framework. Our results are promising and should be validated by many case studies. This is a possibility for other studies worldwide that provide yield information for different crops. This study can also develop different forecast regression models that use spatio-temporal data as predictors.

## Figures and Tables

**Table 1:** Subregions generated from VCI/TCI data and sICA technique.

VCI ICs	Subregions	TCI ICs	Subregions
VCI IC1	MRD	TCI IC1	Southeast + MRD
VCI IC2	Northwest	TCI IC2	Northwest
VCI IC3	Highlands + Southeast	TCI IC3	NCC
VCI IC4	Northeast + RRD	TCI IC4	Northeast+RRD
VCI IC5	NCC + SCC	TCI IC5	SCC + Highlands

**Table 2:** Subregions based on combining the VCI and TCI spatial patterns from Figure 4.

No.	Subregions	VCI ICs	TCI ICs
1	Northwest	VCI IC2	TCI IC2
2	Northeast	VCI IC4	TCI IC4
3	RRD	VCI IC4	TCI IC4
4	NCC	VCI IC5	TCI IC3
5	SCC	VCI IC5	TCI IC5
6	Highlands	VCI IC3	TCI IC5
7	Southeast	VCI IC3	TCI IC1
8	MRD	VCI IC1	TCI IC1
